# Test of the Bifactor Model of Job-Related Affective Well-Being

**DOI:** 10.5964/ejop.v15i2.1632

**Published:** 2019-06-07

**Authors:** Mariola Laguna, Emilia Mielniczuk, Wiktor Razmus

**Affiliations:** a Institute of Psychology, The John Paul II Catholic University of Lublin, Lublin, Poland; Department of Psychology, Webster University Geneva, Geneva, Switzerland; University of Bari "A. Moro", Bari, Italy

**Keywords:** affect, well-being, bifactor model, employees, entrepreneurs

## Abstract

The multidimensional measure of the job-related affective well-being developed by Warr (1990) is a frequently used tool estimating affect in the work context. Alternative factorial models of this measure were tested in previous studies. Recently a bifactor model has been proposed as alternative factorial structure recommended for multifaceted constructs. It allows capturing the global aspect of the construct along with the specificity of its subdimensions. We conducted two studies to test a bifactor model on Warr’s measure and to compare it to factor models proposed in earlier studies. This bifactor model identified one general factor in addition to four unique factors. Two studies were conducted among employees (Study 1; N = 869) and entrepreneurs (Study 2; N = 204). Results of both studies corroborate a four correlated factors model as superior to the bifactor model. The model with four unique but correlated factors representing anxiety, comfort, depression, and enthusiasm is a good representation of job-related affective well-being measured by Warr’s instrument, both in a sample of employees and entrepreneurs.

Affective dimensions of work have become an important research area in work and organizational psychology (Brief & Weiss, 2002; Mäkikangas, Hyvönen, Leskinen, Kinnunen, & Feldt, 2011; Seo, Barrett, & Bartunek, 2004), as well as in the psychology of entrepreneurship (Baron, 2008; Gorgievski & Stephan, 2016; Stephan, 2018). Studies have shown that people’s affective states play a central role in explaining links between work environments, employees behavioral responses and work performance (Parker, 2014). With the increased interest in job-related affective well-being comes a renewed interest in the validity of its measurement instruments. It is vital to accurately measure individuals’ affective experiences, in order to reach valid conclusions for explaining their role in a work environment (Mäkikangas, Feldt, & Kinnunen, 2007).

One of the most widely used tools for testing affect in the work context is Warr’s (1990) job-related affective well-being measure (Laguna, Mielniczuk, Razmus, Moriano, & Gorgievski, 2017; Mäkikangas et al., 2007; Salanova, Llorens, & Schaufeli, 2011; Van Horn, Taris, Schaufeli, & Schreurs, 2004). As Warr has not proposed a name for this measure, authors customarily refer to it as “Warr’s (1990) job-related affective well-being measure,” “the measure developed by Warr (1990),” or like phrases. This name may seem similar to the name of the Job-Related Affective Well-Being Scale (JAWS) developed by Van Katwyk et al. (2000), however, these are two different instruments. To clarify, JAWS is also based on Warr’s model of affective well-being at work, which was primarily operationalized by his own measure. Previous analyses (Daniels, Brough, Guppy, Peters-Bean, & Weatherstone, 1997; Gonçalves & Neves, 2011; Mäkikangas et al., 2007; Sevastos, Smith, & Cordery, 1992) have confirmed the validity and reliability of the instrument developed by Warr (1990).

What has not been tested, however, is the validity of a potential bifactor model, which has recently been postulated as a recommended alternative method for testing multifaceted constructs (Chen, Hayes, Carver, Laurenceau, & Zhang, 2012). Research has demonstrated that bifactor models which identify one general factor in addition to multiple unique factors, allow the superior representation of different personality constructs (Alessandri, Vecchione, Eisenberg, & Łaguna, 2015; Chen, Watson, Biderman, & Ghorbani, 2016), as well as of well-being (Chen, Jing, Hayes, & Lee, 2013) and affect (Leue & Beauducel, 2011). Such bifactor representation allows capturing the global aspect of the construct along with the specificity of its subdimensions. This suggests that a bifactor model may be a better representation of job-related affect than previously tested models. Moreover, for some research purposes, partialing out the general factor may be a preferred way of analyzing data. In the case of well-being, we can expect that such a general factor may be influenced by stable, internal processes rather than momentary, environmental factors.

Therefore, the aim of our research was to determine whether a bifactor model may represent properly, and better than alternative models, job-related affective well-being as captured by the measure developed by Warr (1990). Our research tested the factorial structure of this measure not only on a sample of employees, but also, for the first time, on a sample of entrepreneurs – people who established themselves and manage their own firms (Baron, 2007). Using relatively large, diverse samples, and a sound analytic framework, we were able to comprehensively test the factorial structure of the widely-used measure proposed by Warr (1990) and provide recommendations how the results can be interpreted in future studies and counseling practices.

## Job-Related Affective Well-Being Measure

Affect as consciously accessible feelings evident in moods and emotions (Fredrickson, 2001; Walter & Bruch, 2008) is a vital sign of an individual’s subjective well-being (Chen, Jing, et al., 2013). It also constitutes the most central dimension of occupational subjective well-being (Van Horn et al., 2004) which has become of interest in work and organizational psychology (Mäkikangas et al., 2011; Seo et al., 2004), in career counseling (Cuyper, Philippaers, Vanhercke, & Witte, 2017; Gati et al., 2011; Hutchison & Gerstein, 2017; Konstam, Celen-Demirtas, Tomek, & Sweeney, 2015), and in entrepreneurship (Stephan, 2018). There is growing evidence which shows that employees’ job-related affect is linked to their work engagement (Bledow, Schmitt, Frese, & Kühnel, 2011; Salanova et al., 2011), direction, intensity, and persistence of actions (Seo et al., 2004). Affect also impacts performance at work (Beal, Weiss, Barros, & MacDermid, 2005), as well as satisfaction from performed tasks and responsibility for those tasks (Isen & Reeve, 2005). Affect is important not only in the activity of employees, but also that of entrepreneurs (Baron, 2008; Stephan, 2018) and this is related to their work engagement and goal attainment (Laguna, Alessandri, & Caprara, 2016; Laguna, Razmus, & Żaliński, 2017). All these findings show that job related affect should be taken into account when discussing career choices, job satisfaction and performance.

This broad interest in job-related affective well-being raises the question of how to accurately estimate it to represent the structure of an individual’s emotional experience (Mäkikangas et al., 2007). A conceptualization of affective well-being, accompanied by a measurement tool, was proposed by Warr (1990). The measure is based on the circumplex model of affect (Russell, 1980) and conceptualizes well-being as a job-specific rather than as a context-free phenomenon. According to Warr (1990), job-related affective well-being is defined by two principal dimensions: pleasure (negative—positive) and arousal (low—high activation level). The measure is intended to capture the content and intensity of job-related emotional states: Comfort (positive affect of low arousal), Enthusiasm (positive affect of high arousal), Depression (negative affect of low arousal), and Anxiety (negative affect of high arousal).

The instrument consists of 12 adjectives (e.g., calm, worried) which are answered in relation to the job: “Thinking of the past few weeks, how much of the time has your job made you feel each of the following?” It is also possible to measure context-free well-being with different phrasing of the instruction.

## The Factorial Structure of the Job-Related Affective Well-Being Measure

The factorial structure of the measure proposed by Warr (1990) was assessed in a several studies (Cifre & Salanova, 2002; Daniels et al., 1997; Gonçalves & Neves, 2011; Laguna, Mielniczuk, et al., 2017; Mäkikangas et al., 2007; Mielniczuk & Laguna, 2018; Sevastos et al., 1992; Van Horn et al., 2004; Warr, 1990) in which six alternative models were tested (for details see Appendix). Model 1 contained four correlated factors that were postulated in Warr’s model (1990): Anxiety, Comfort, Depression, and Enthusiasm. Model 2 posited two correlated factors, capturing two dimensions: Anxiety–Comfort and Depression–Enthusiasm. Model 3 posited two correlated factors: Positive Affect and Negative Affect. Model 4 represented three dimensions: Positive Affect, Negative Affect, and Pleasantness–Unpleasantness. Model 5 was a single factor model capturing affective well-being as a single common factor. The hierarchical Model 6 captured the Global Affective Well-being as a second-order factor and four first-order latent factors.

Investigations of the general factor of job-related affective well-being (Gonçalves & Neves, 2011; Van Horn et al., 2004) raise the question of how well-being should be understood: Does it primarily refer to specific affective dimensions, or should it be considered as a more global phenomenon that captures the general subjective estimation of whether a person feels well or unwell at work? Both conceptualizations are of interest in counselling and in work and organizational psychology research.

A potential solution may be the consideration of a bifactor model as an alternative to the single factor or hierarchical models (Chen, Hayes, et al., 2012). The bifactor model can be considered when (a) there is a general factor accounting for the commonality of the items; (b) there are multiple domain specific factors; and (c) the common factor together with the domain-specific factors can be interesting for researchers (Chen, Hayes, et al., 2012). This proposition was validated as a valuable and unsurpassed alternative in explaining dimensionality of personality traits (Chen et al., 2016), self-esteem (Alessandri et al., 2015), context-free well-being (Chen, Jing, et al., 2013), context-free affect (Leue & Beauducel, 2011). This would suggest that the bifactor model may be a better estimate of job-related affective well-being than previously tested models.

**Figure 1:**
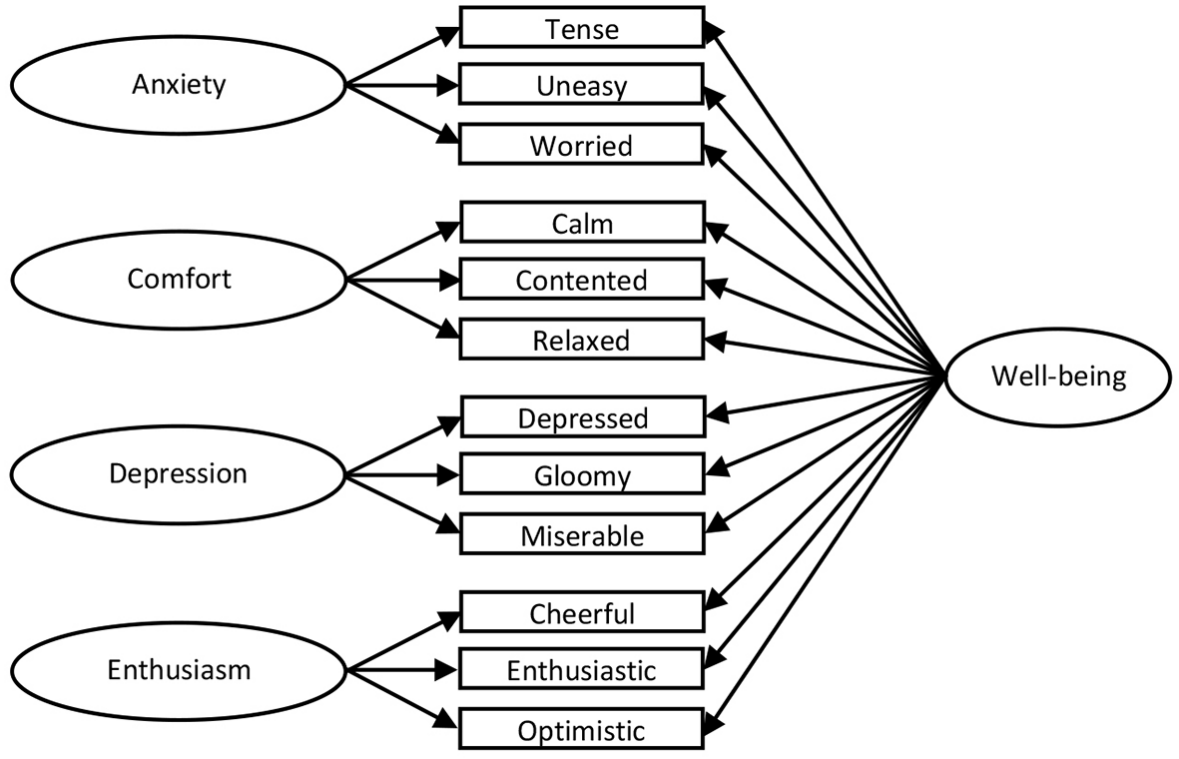
The bifactor model for the Warr’s (1990) measure (Model 7).

Hence, we propose a new model: Model 7 as a bifactor representation of job-related affective well-being (Figure 1). It captures the global phenomenon of job-related well-being in addition to specific affective dimensions: Anxiety, Comfort, Depression, and Enthusiasm. Model 7 is proposed as an alternative way of representing the core features of an underlying global latent dimension than a hierarchical model (Gonçalves & Neves, 2011) or a single factor model (Van Horn et al., 2004).

An attractive feature of this bifactor interpretation is that it casts the specific factors in a manner consistent with previous empirical research that confirmed four specific affective well-being dimensions from Warr’s item pool. However, the simple procedure of summing the items representing each dimension left room for possible contamination of the scale scores, since variance from general job-related affective well-being was not partialled-out from the scores. This problem can be avoided by the bifactor approach (Chen, Hayes, et al., 2012). Nevertheless, even if successfully applied towards other measures (Alessandri et al., 2015; Chen et al., 2016; Leue & Beauducel, 2011), it is not guaranteed that this approach will be fruitful in estimating emotional experiences at work. Therefore, the aim of our research was to validate the bifactor model on Warr’s instrument (1990) in comparison to alternative models established in previous studies. The alternative measurement models considered in our research are depicted in Appendix.

We conducted two studies to investigate the relative fit of seven alternative factor models described above, including newly proposed the bifactor model. In both studies, we relied on relatively large samples which allowed us to use Structural Equation Modeling (SEM) in order to address several important psychometric questions pertaining to the assessment of affective well-being. We focused on the dimensionality of the instrument, based on the accepted methodological procedures (Chen, Hayes, et al., 2012). The relative fit of the hypothesized alternative models was iteratively tested in samples of employees (Study 1) and entrepreneurs (Study 2).

## Study 1

The aim of this study was to examine the relative fit of seven alternative models, attesting the factorial structure of the job-related affective well-being measure (Warr, 1990) on a sample of employees.

### Method

#### Procedure

The data was gathered using the paper-and-pencil questionnaires (62.5% of the sample) and on-line questionnaires (37.5%). In additional analyses, measurement invariance for the sampling method was tested and full scalar invariance was achieved. Participants were invited to take part in the study through social and business networks using direct solicitation. Paper-and-pencil questionnaires were delivered to participants at their homes or at work and collected from them after completion. Participation in the study was voluntary and the participants did not receive any reward. The confidentiality and anonymity of data was ensured. We computed the minimum sample size for the model structure (*N* = 187) as a function of the required significance level of α = .05, and the desired statistical power of 1-β = .80.

#### Participants

The sample included 818 employees from Poland, of whom 59.5% were women, and it far exceeded the minimum sample size. The participants were 19 to74 years old (*M* = 33.92, *SD* = 10.13), and worked full time (61.8%), part time (30.8%), or on other forms of work contracts (7.3%). They worked in services and trade (45%), production (15.1%), construction (1.7%), or in other sectors (38.2%). The sample generally matched the characteristics of the population of employees in Poland, except that it included a few more women (54.9% men and 45.1% of woman in the population; GUS 2017).

#### Measure

Job-related affective well-being was evaluated using the Polish version (Mielniczuk & Laguna, 2018) of the measure proposed by Warr (1990). It consisted of 12 items and answers were given on a 6-point scale (1 = *never*; 2 = *occasionally*; 3 = *some of the time*; 4 = *much of the time*; 5 = *most of the time*; 6 = *all of the time*).

#### Data Analysis Strategy

The data were analyzed by means of confirmatory factor analysis (CFA) using Amos (Arbuckle, 2005). The parameters were estimated using the maximum likelihood method. Missing data, which did not exceed 1.5% in any item, were handled using regression imputation.

We examined a series of seven measurement models. To assess model fit, we used the χ^2^ Goodness-of-Fit Statistic, the Comparative Fit Index (CFI), the Root Mean Square Error of Approximation (RMSEA), the Standardized Root Mean Square Residual (SRMR), and the Akaike Information Criterion (AIC). Values below .08 for RMSEA and SRMS indicate an acceptable model fit (Kline, 2005), while values below .10 indicate mediocre model fit (Hooper, Coughlan, & Mullen, 2008). For the CFI values greater than .90 indicate a good fit (Brown, 2006; Hooper et al., 2008). The lower the AIC index, the better is the fit of the model to the data (Brown, 2006). To test differences between alternative models, we calculated the chi-square difference tests (Δχ^2^) and difference in CFI (ΔCFI; Brown, 2006; Kline, 2005). An absolute difference in CFI that is less than .01 (ΔCFI < .01) would indicate a lack of significant difference in model fit (Cheung & Rensvold, 2002).

### Results and Discussion

Examination of alternative models showed that only the four correlated factors model (Model 1) obtained acceptable fit in all fit indices (Table 1).

None of the alternative models reached the criteria of acceptable fit (Brown, 2006; Kline, 2005), with the exception of SRMR for a bifactor model (SRMR = .069), which is inconsistent with other fit indices. Moreover, tests of differences (Δχ^2^ < .05; ΔCFI > .01) confirmed that the four correlated factors model fit the data significantly better than any other alternative model, including the bifactor model (lower χ^2^ value and ΔCFI > .01). The bifactor solution is therefore not confirmed in this study.

**Table 1:** Goodness-of-Fit Indices of Alternative Measurement Models in Sample of Employees (N = 869)

Model	χ^2^	*df*	*p*	RMSEA	SRMR	CFI	AIC	Model comparison	Δχ^2^	Δ*df*	*p*	ΔCFI
Model 1. Four correlated factors: Anxiety, Comfort, Depression, Enthusiasm	286.04	48	< .001	.076	.043	.960	370.04	-	-	-	-	-
Model 2. Two correlated factors: Anxiety-Comfort, Depression-Enthusiasm	1560.36	53	< .001	.181	.123	.748	1610.36	M7 vs M1	1274.32	5	< .001	.21
Model 3. Two correlated factors: Positive Affect, Negative Affect	979.14	53	< .001	.142	.083	.845	1029.14	M2 vs M1	693.10	5	< .001	.12
Model 4. Three factors: Positive Affect, Negative Affect, Pleasantness-Unpleasantness	1270.98	51	< .001	.166	.107	.796	1324.98	M8 vs M1	984.94	3	< .001	.16
Model 5. Single factor: Well-being	1855.53	54	< .001	.196	.116	.699	1903.53	M4 vs M1	1569.49	6	< .001	.26
Model 6. Hierarchical: four factors and higher order factor Well-being	417.98	50	< .001	.092	.073	.939	473.98	M5 vs M1	131.94	2	< .001	.02
Model 7. Bifactor: four factors and Well-being	372.89	48	< .001	.095	.069	.945	468.89	M6 vs M1	86.85	0	< .001	.02

*Note.* RMSEA = Root Mean Square Error of Approximation; SRMR = Standardized Root Mean Square Residual; CFI = Comparative Fit; AIC = Akaike Information Criterion.

In this best fitting four correlated factors model (Model 1) regression weights were high, varied between .87 and .69 for each of four latent factors. Descriptive statistics, reliability and correlations between scales are presented in Table 2. Cronbach’s alpha range from .78 to .90 indicated satisfactory to high internal consistency of all scales.

**Table 2:** Scales Reliability, Descriptive Statistics and Correlations Between Scales in Samples of Employees (Study 1, N = 869) and Entrepreneurs (Study 2, N = 204)

Employees	α	*M*	*SD*	1	2	3	Entrepreneurs	α	*M*	*SD*	1	2	3
1. Anxiety	.84	2.89	1.01				1. Anxiety	.82	2.71	.79			
2. Comfort	.83	3.53	1.00	-.61			2. Comfort	.78	3.25	.71	-.54		
3. Depression	.79	2.12	0.92	.55	-.48		3. Depression	.81	1.60	.59	.67	-.53	
4. Enthusiasm	.89	3.81	1.09	-.39	.69	-.40	4. Enthusiasm	.90	4.27	.92	-.35	.78	-.40

*Note.* All correlations are statistically significant at the level of at least *p* < .01 (two-tailed).

In conclusion, the results of this study demonstrated that the model representing job-related affective well-being as comprised of four correlated dimensions is better representation of emotional experiences of employees than the bifactor model.

## Study 2

In Study 2, the bifactor model was again compared with alternative models of job-related affective well-being (Warr, 1990) on a sample of entrepreneurs. This allowed us to check whether the findings from Study 1 on employees could be generalized to other samples. As entrepreneurs, we consider people who established and manage their firms themselves (Baron, 2007), and were, therefore, not only managers, but also new organization builders.

### Method

#### Procedure

The snowball sampling method was used where the initially-contacted entrepreneurs from different regions of Poland were asked to provide contacts to two other entrepreneurs. The participants were invited to take part in the study if they fulfilled four criteria, being: (1) founders, (2) owners, and (3) managers of their firms, and (4) had firms that survived on the market for at least one year. The respondents were asked to fill in the paper-and-pencil questionnaires. Participation in the study was voluntary and the participants did not receive any reward. The data were anonymized to ensure confidentiality. The minimum sample size (*N* = 187) was calculated similarly as in Study 1.

#### Participants

A sample of 204 entrepreneurs took part in the study, of whom 123 were male (61.9%). It exceeded the minimum sample size. The participants were 22-71 years old (*M* = 44.07, *SD* = 11.53). The firms they managed had existed for between one and 47 years (*M* = 10.10, *SD* = 8.36). Sectors of operation included services (79.9%), production (11.3%), and construction (8.8%). All of these firms were small and medium-sized enterprises, employing up to 82 employees for full-time contracts (*M* = 6.29, *SD* = 13.12), and up to 32 employees for part-time contracts (*M* = 1.55, *SD* = 3.43). The sample matched the characteristics of a population of entrepreneurs in Poland (Tarnawa & Zadura-Lichota, 2013).

#### Measure

Job-related affective well-being was assessed using the Polish version (Mielniczuk & Laguna, 2018) of the measure proposed by Warr (1990).

#### Data Analysis Strategy

We used Amos (Arbuckle, 2005) to perform SEM analyses; the maximum likelihood estimation was applied. Missing data, not exceeding 1.5% in any item, were handled using the regression imputation method. CFA was applied to examine alternative models.

### Results and Discussion

To further validate the results of Study 1, we examined the seven alternative models again. CFA results (Table 3) show that the model with four correlated factors (Model 1) obtained an acceptable fit in all fit indices (χ^2^_(48)_ = 129.601, *p <* .001, CFI = .96, AIC = 213.60, RMSEA = .092, SRMR = .059), except for RMSEA which exceeded the criterion value of .08. Neither the bifactor model nor any of the alternative models reached the acceptable level of model fit. Moreover, the comparison of differences (Δχ^2^ < .05; ΔCFI > .01) between Model 1 and all other alternative models show that it fits the data significantly better. Therefore, again, the bifactor model was not supported.

**Table 3:** Goodness-of-Fit Indices of Alternative Measurement Models in Sample of Entrepreneurs (N = 204)

Model	χ^2^	*df*	*p*	RMSEA	SRMR	CFI	AIC	Model comparison	Δχ^2^	Δ*df*	*p*	ΔCFI
Model 1. Four correlated factors: Anxiety, Comfort, Depression, Enthusiasm	129.601	48	< .001	.092	.059	.956	213.601	-	-	-	-	-
Model 2. Two correlated factors: Anxiety-Comfort, Depression-Enthusiasm	492.626	53	< .001	.202	.152	.657	542.626	M2 vs M1	363.025	5	< .001	.279
Model 3. Two correlated factors: Positive Affect, Negative Affect	263.473	53	< .001	.140	.088	.836	337.473	M3 vs M1	133.872	5	< .001	.100
Model 4. Three factors: Positive Affect, Negative Affect, Pleasantness-Unpleasantness	430.120	51	< .001	.191	.135	.704	484.120	M4 vs M1	300.519	3	< .001	.232
Model 5. Single factor: Well-being	548.672	54	< .001	.212	.141	.614	596.672	M5 vs M1	419.071	6	< .001	.322
Model 6. Hierarchical: four factors and higher order factor Well-being	162.704	50	< .001	.105	.099	.912	218.704	M6 vs M1	33.103	2	< .001	.024
Model 7. Bifactor: four factors and Well-being	138.421	42	< .001	.106	.085	.925	234.421	M7 vs M1	8.820	2	.012	.011

*Note*. RMSEA = Root Mean Square Error of Approximation; SRMR = Standardized Root Mean Square Residual; CFI = Comparative Fit; AIC = Akaike Information Criterion.

Regression weights for each of the four correlated factors varied between .86 and .62. Descriptive statistics and correlations between scales are presented in Table 2. Cronbach’s alpha for the four scales ranged between .78 and .90, indicating satisfactory to high internal consistency of four scales.

## Discussion

A bifactor solution to factorial structure of measures was recently postulated for testing multifaceted constructs (Chen, Hayes, et al., 2012). Such bifactor models allow to identify a general factor in addition to multiple unique factors, which are of interest in personality psychology (Alessandri et al., 2015; Chen et al., 2016), and in well-being research (Chen, Jing, et al., 2013; Leue & Beauducel, 2011). We expected that such bifactor model may be also a good representation of job-related affective well-being. Results of our two studies, however, did not confirm this expectation. Our research findings supported the four correlated factors model over the proposed bifactor model. According to our results, neither the bifactor, nor a single factor or hierarchical model fit the data better than the four factor model; this is consistent with the results of other studies (Gonçalves & Neves, 2011; Laguna, Mielniczuk, et al., 2017; Mäkikangas et al., 2007; Mielniczuk & Laguna, 2018). All of those findings confirm that job-related affective well-being, as measured by Warr’s instrument (1990) capture four specific, interrelated affective dimensions. This altogether supports the job-related affective well-being dimensionality, and subsequently, the circumplex model of affect (Russell, 1980). It postulates that each affective experience is the result of a combination of two independent neurophysiological systems of valence and arousal, and is then interpreted as representing rather a particular affects (Posner, Russell, & Peterson, 2005), and not a global affectivity. Therefore, even if in case of self-esteem (Alessandri et al., 2015), context-free well-being (Chen, Jing, et al., 2013), context-free affect (Leue & Beauducel, 2011) the global aspect of the construct along with the specificity of its subdimensions can be distinguished, in case of job-related affective well-being (as measured by the Warr instrument) a global affectivity dimension is not confirmed.

### Strengths and Limitations

One of the strengths of this research lies in the variation of samples included in our two studies. We involved a large sample of employees of different professions and, for the first time, made an evaluation of psychometric properties of the measure on the sample of entrepreneurs. This validates the instrument as useful in research on the role of affect in entrepreneurship – a dynamically evolving field of study (Baron, 2008; Laguna et al., 2016; Stephan, 2018). Another strong point of this study is that we tested a broad spectrum of alternative structural models. We checked all potential models tested in previous analyses and added a new, theoretical and psychometric proposition of the bifactor model (Chen, Hayes, et al., 2012).

The studies were, however, not free of some shortcomings. First, our analyses were based on samples coming from one country, Poland. Even if the factorial structure of the measure was confirmed in other samples of Polish employees (different than one presented in this paper, i.e., employees of small and medium size companies Laguna, Mielniczuk, et al., 2017; Mielniczuk & Laguna, 2018) and shows cross-culture invariance (Laguna, Mielniczuk, et al., 2017; Mielniczuk & Laguna, 2018), the bifactor model should be examined further in other cultural contexts.

### Conclusions

In conclusion, our results suggest that in future analyses four job-related affective well-being dimensions of the Warr (1990) measure should be applied rather than the bifactor model. The four scales show satisfactory reliability in samples of employees of different profession and of entrepreneurs.

As work activity constitutes one of the central domains of functioning for most adults, job-related affect is a vital part of the human experience which should be taken into account when dealing with problems of people seeking professional help. This may be essential in career guidance and counseling where there is also an increasing focus on the emotional functioning of clients (Gati et al., 2011). Knowledge on what kind of emotions a client experiences toward work may help to prepare interventions targeted at specific problems. This can be done through the use of different affect-related interventions (Koydemir & Sun-Selisik, 2016; Saedpanah, Salehi, & Moghaddam, 2016). Warr’s (1990) measure can be applied as a useful tool for evaluating affective experiences related to work and distinguishing four affective dimensions differing in valence (i.e., positive and negative affect) and activation level rather than a single global affectivity dimension.

## Figures and Tables

**Figure A.1:**
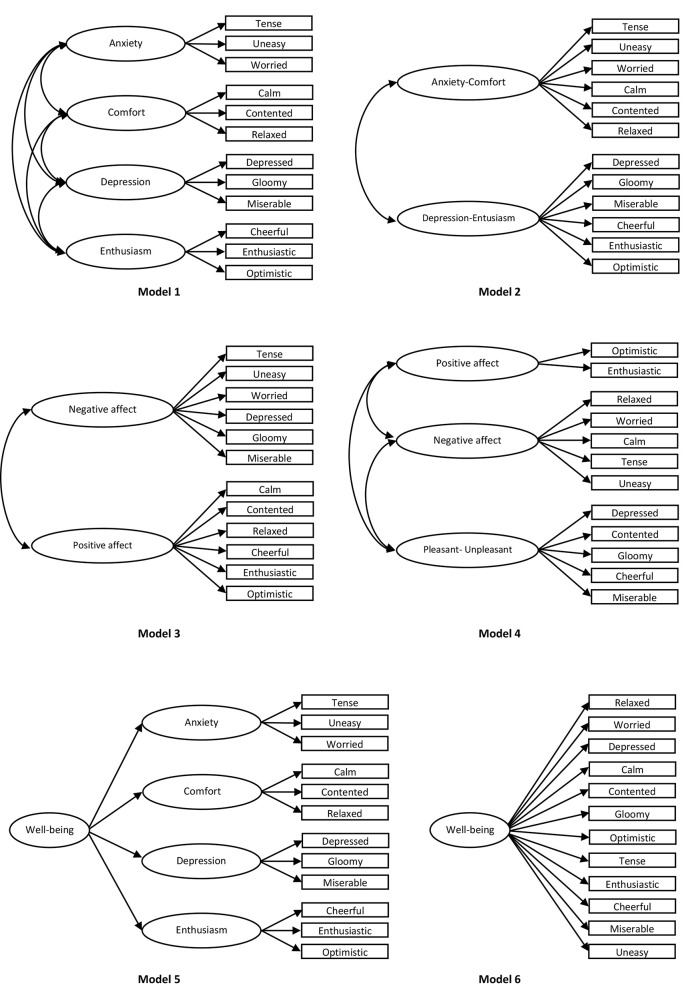
Six alternative measurement models for the Warr’s (1990) measure.
